# Whole genome sequencing of SIV-infected macaques identifies candidate loci that may contribute to host control of virus replication

**DOI:** 10.1186/s13059-014-0478-z

**Published:** 2014-11-07

**Authors:** Adam J Ericsen, Gabriel J Starrett, Justin M Greene, Michael Lauck, Muthuswamy Raveendran, David Rio Deiros, Mariel S Mohns, Nicolas Vince, Brian T Cain, Ngoc H Pham, Jason T Weinfurter, Adam L Bailey, Melisa L Budde, Roger W Wiseman, Richard Gibbs, Donna Muzny, Thomas C Friedrich, Jeffrey Rogers, David H O’Connor

**Affiliations:** Department of Pathology, University of Wisconsin-Madison, Madison, WI 53705 USA; Virology Training Program, University of Wisconsin-Madison, Madison, WI 53705 USA; Human Genome Sequencing Center, Baylor College of Medicine, Houston, TX 77030 USA; Department of Pathobiological Sciences, University of Wisconsin-Madison, Madison, WI 53706 USA; Cancer and Inflammation Program, Laboratory of Experimental Immunology, Leidos Biomedical Research Inc., Frederick National Laboratory for Cancer Research, Frederick, MD 21701 USA; Ragon Institute of MGH, MIT and Harvard, Cambridge, MA 02139 USA; Wisconsin National Primate Research Center, Madison, WI 53715 USA; 585 Science Drive, Madison, WI 53711 USA

## Abstract

**Background:**

A small percentage of human immunodeficiency virus (HIV)-infected people and simian immunodeficiency virus (SIV)-infected macaques control virus replication without antiretroviral treatment. The major determinant of this control is host expression of certain major histocompatibility complex alleles. However, this association is incompletely penetrant, suggesting that additional loci modify the major histocompatibility complex’s protective effect. Here, to identify candidate control-modifying loci, we sequence the genomes of 12 SIV-infected Mauritian cynomolgus macaques that experienced divergent viral load set points despite sharing the protective M1 major histocompatibility complex haplotype.

**Results:**

Our genome-wide analysis of haplotype-level variation identifies seven candidate control-modifying loci on chromosomes 2, 3, 7, 8, 9, 10, and 14. The highest variant density marks the candidate on chromosome 7, which is the only control-modifying locus to comprise genes with known immunological function. Upon closer inspection, we found an allele for one of these genes, granzyme B, to be enriched in M1(+) controllers. Given its established role as a cytotoxic effector molecule that participates in CD8-mediated killing of virus-infected cells, we test the role of variation within *gzmb* in modifying SIV control by prospectively challenging M1(+) granzyme B-defined macaques.

**Conclusions:**

Our study establishes a framework for using whole genome sequencing to identify haplotypes that may contribute to complex clinical phenotypes. Further investigation into the immunogenetics underlying spontaneous HIV control may contribute to the rational design of a vaccine that prevents acquired immune deficiency syndrome.

**Electronic supplementary material:**

The online version of this article (doi:10.1186/s13059-014-0478-z) contains supplementary material, which is available to authorized users.

## Background

The majority of HIV-infected individuals experience progressive infection, characterized by ongoing virus replication, CD4(+) cell depletion, and eventually acquired immune deficiency syndrome (AIDS). However, a rare group of infected individuals spontaneously suppress virus replication to low or undetectable levels during chronic infection [1,2]. Although the basis for this control is likely multifaceted, certain major histocompatibility complex (MHC) class I alleles, such as human leukocyte antigen (HLA)-B*27 and HLA-B*57, are overrepresented among controllers [3], and genome-wide association studies (GWASs) indicate that the MHC is the dominant genetic correlate of chronic phase control of viral replication [1]. Similarly, in macaque monkeys infected with pathogenic SIV, control of viremia is strongly influenced by MHC genotypes (reviewed in [4]). However, not all infected individuals with protective MHC genetics become controllers [5], and understanding the host parameters responsible for this incomplete penetrance is a major priority for HIV/AIDS research.

Despite the overall complexity of the host response to HIV/SIV, there is consensus that CD8(+) T cells are primary mediators of sustained virus control [6,7]. Multiple studies have sought to identify key features of the HIV-specific CD8(+) T cells underlying the variability in chronic phase viral suppression. Compared with cells from progressors, CD8(+) T cells isolated from controllers have been shown to preferentially target specific viral proteins [8,9], express a greater number of cytokines following *in vitro* peptide stimulation [10–12], exhibit enhanced proliferative potential [10,13,14], and rapidly upregulate and release cytotoxic granule proteins upon encountering cognate MHC-peptide complexes on the surface of infected cells [13,15,16]. Despite these functional correlates, compelling host parameters have not yet been identified to explain why these immunological differences exist between controllers and progressors.

Within the past half-millennia, a small founder population of cynomolgus macaques was deposited onto the island of Mauritius [17] and expanded, in geographic isolation, to constitute what is now a population of several thousand animals. Due to founder effects, even highly polymorphic loci, such as the MHC [18–20] and killer immunoglobulin receptor (KIR) [21], are represented by fewer than 10 extended haplotypes. This highly restricted genetic diversity makes Mauritian cynomolgus macaques (MCMs) an ideal animal model with which to interrogate the role of genetic variation in complex clinical phenotypes.

Sequencing large and complex eukaryotic genomes is becoming increasingly accessible [22–25], and its incorporation into HIV infection and SIV challenge studies is now practical. Here, to begin to identify genetic variation outside of the MHC that modifies control of HIV/SIV in individuals possessing protection-associated MHC alleles, we sequenced the whole genomes of 12 SIV-infected MCMs (six controllers and six progressors) that shared the MHC M1 haplotype, which is the most common of seven simple MHC haplotypes (M1 to M7) in MCMs. The M1 haplotype was defined using short tandem repeat-based typing across a 5 Mb region of macaque chromosome 4 [20], and it encodes at least three MHC class I alleles that altogether restrict eight SIV-specific CD8(+) T-cell responses [26]. Recently, M1 was found to be enriched in, but not exclusive to, macaques that control SIV [9]. Thus, the incomplete penetrance of M1-associated SIV control provides a tractable non-human primate model with which to identify and interrogate candidate minor determinants, or modifiers, of MHC-associated AIDS virus control.

## Results

### Whole genome sequencing of SIV-infected Mauritian cynomolgus macaques

**Figure 1:**
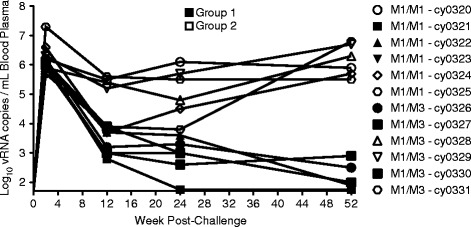
**Longitudinal viral loads for Cohort A.** Longitudinal viral load plot for 12 M1(+) Mauritian cynomolgus macaques. Shaded symbols indicate Group 1 animals (controllers) and open symbols indicate Group 2 animals (progressors).

We retrospectively assembled a cohort (Cohort A) of 12 M1(+) male MCMs (Table 1), six M1/M1 animals and six M1/M3 animals that were infected with SIVmac239 as part of a previous study [9]. All animals used in this study were SIV-naïve prior to experimental challenge, and did not receive any antiviral intervention during the post-challenge period of observation. For all downstream analyses, control in Cohort A animals was defined as suppression of peripheral viremia (viral load) to below 1,000 viral RNA copies per milliliter of blood plasma (or 3Log_10_). Although SIV viremia began to diverge within 12 weeks following challenge (Figure 1), Cohort A viral loads did not sufficiently stabilize to clearly differentiate durable controllers (Group 1) from progressors (Group 2) until approximately 52 weeks post-challenge.

**Table 1:** **Animals used for whole genome sequencing**

**Designation**		**ID**	**MHC**	**GZMB**	**52 week viral load (log10)**	**Sex**	**Number of reads mapped**	**Reference**
Cohort A	Group 1	cy0321	M1/M1	G1/G5	1.7	M	1222977688	[9]
		cy0322	M1/M1	G1/G4	1.9	M	1093240092	[9]
		cy0323	M1/M1	G1/G3	2.0	M	1161761415	[9]
		cy0326	M1/M3	G1/G4	2.5	M	1014048607	[9]
		cy0327	M1/M3	G1/G5	2.0	M	1158978497	[9]
		cy0330	M1/M3	G3/G6	2.9	M	1120820126	[9]
	Group 2	cy0320	M1/M1	G4/G4	5.9	M	1210339744	[9]
		cy0324	M1/M1	G4/G4	5.7	M	1192434815	[9]
		cy0325	M1/M1	G2/G3	6.8	M	1091870521	[9]
		cy0328	M1/M3	G4/G4	6.3	M	1356197360	[9]
		cy0329	M1/M3	G2/G3	6.7	M	1169431587	[9]
		cy0331	M1/M3	G4/G5	5.5	M	1314518723	[9]
Outgroup		cy0332	M3/M3	G2/G4	6.1	M	1206633787	[9]
		cy0333	M3/M3	G3/G5	6.5	M	1217425129	[9]
		cy0334	M3/M3	G2/G5	5.0	M	1094844832	[9]
		cy0335	M3/M3	G1/G4	5.0	M	1298840412	[9]
		cy0336	M3/M3	G2/G5	6.2	M	1244446132	[9]
		cy0337	M3/M3	G3/G4	6.0	M	1357235243	[9]

Whole genome sequencing was performed on 18 SIV-infected Mauritian cynomolgus macaques. For each animal, the MHC genotype, GZMB genotype, 52 week viral load (log_10_ viral RNA copies per milliliter of plasma), sex, and number of sequencing reads mapped to rheMac2 are shown for the 12 Cohort A M1(+) animals, and an outgroup of 6 M3/M3 macaques.

In order to better understand the incomplete penetrance of M1-associated control, we used the Illumina HiSeq platform and 90 bp paired-end reads to sequence the genomes of all 12 Cohort A animals, as well as six additional animals described in [9]. On average, 1.2 billion reads for each animal were mapped to the rheMac2 rhesus macaque (*Macaca mulatta*) reference genome (NCBI assembly Mmul_051212). After mapping, these genomes had an average of 35-fold coverage with 8.5% of the genome having no coverage, which may have been a consequence of mapping cynomolgus macaque (*Macaca fascicularis*) sequences to the rhesus macaque reference genome, large gaps within the reference genome, difficulty in sequencing GC-rich regions, or difficulties inherent to mapping complex repeat regions. Individual animal mapping coverage is shown in Table 1, and per chromosome coverage for each animal in Cohort A is shown in Additional file 1. Homozygous variants common to all animals were considered to be differences between the MCM population overall and rheMac2 reference, and were excluded from our analysis. Only polymorphic sites meeting a quality threshold of at least 30 (Q ≥30) and covered by at least 10 reads in all 18 samples were included in the downstream analyses (a total of 22,526,976 bp).

### Assessing MHC identity in M1(+) macaques

**Figure 2:**
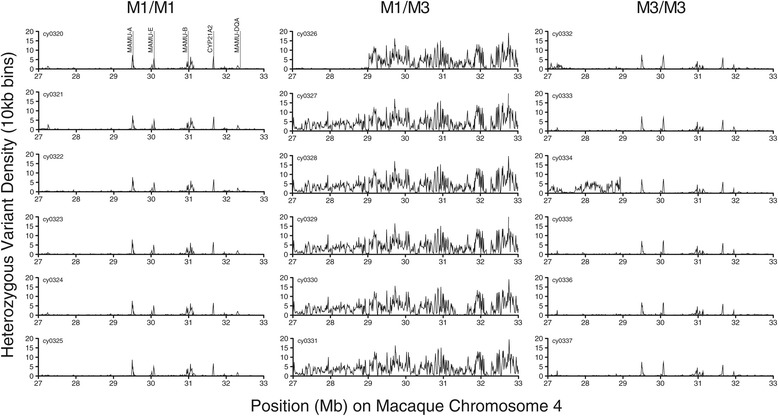
**Density of heterozygous variation across the MHC.** Plots of heterozygous variant density (using 10 kb bins) across the MHC on macaque chromosome 4. As expected, the density of variants is higher, and more widespread, in the M1/M3 heterozygotes compared with both the M1 and M3 homozygotes. As shown in the upper leftmost plot, the occasional peaks of variation detected in the M1 and M3 homozygotes correspond to polymorphic loci upon which reads for multiple alleles mapped.

We first asked whether whole genome sequencing could be used to distinguish MHC-haplotype-identical animals from one another by polymorphisms occurring within the MHC on macaque chromosome 4. To test this, we plotted the density of heterozygous variation across the MHC using 10 kb bins (Figure 2). Consistent with results from short tandem repeat-based MHC typing, within the six M1/M3 animals we found widespread heterozygous variation relative to the six M1/M1 and six M3/M3 homozygous animals. As shown in Figure 2, minor peaks of variation were identified in the M1 and M3 homozygotes, which corresponded to highly polymorphic loci, such as the multicopy class I genes *Mafa-A*, *Mafa-E*, and *Mafa-B*, which have undergone complex duplications and are poorly resolved in rheMac2.

### Identifying regions of group-segregating variation

**Figure 3:**
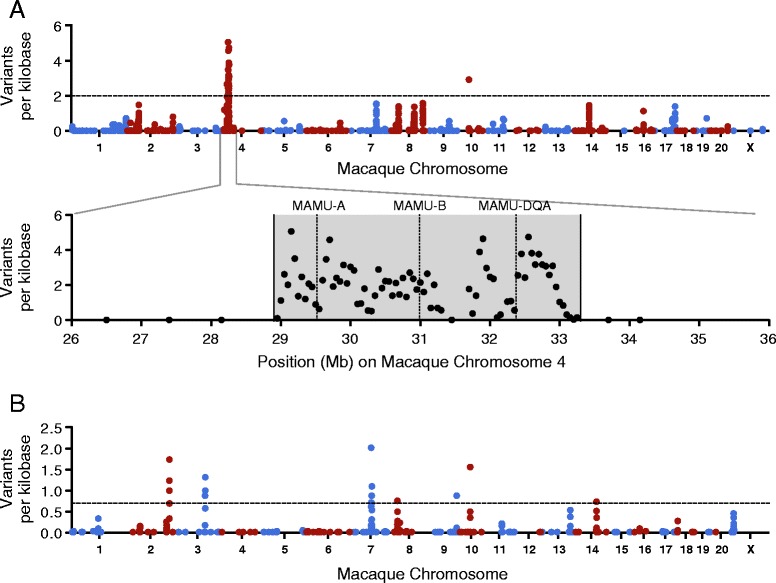
**Variation differentiating MHC homozygotes from heterozygotes and SIV controllers from progressors. (A)** Variants common to six M1/M1 animals, but absent from six M1/M3 animals (and the reciprocal), were identified. Using a Manhattan plot, genome-wide variation was plotted as variants per kilobase across 50 kb bins. Blue and red correspond to alternating chromosome numbers. The highest density of variation across the genome marked the MHC on macaque chromosome 4. Although their annotations do not adequately depict the dispersion of these multicopy loci throughout the MHC, Mamu-A, Mamu-B and Mamu-DQA are shown for reference. **(B)** Variants that strictly segregated between Group 1 (controllers) and Group 2 (progressors) were identified, and the density of this variation was plotted across 21 macaque chromosomes using 50 kb bins.

Our small cohort study is not powered to resolve nucleotide-level variation associated with SIV control. However, by leveraging the restricted genetic diversity of MCM, we postulated that haplotype-level control-associated variation could be used to define candidate control-modifying loci (CMLs) as genomic regions marked by a high-density of individual control-segregating variants. To establish our method, we hypothesized that M1(+) homozygotes could be distinguished from M1(+) heterozygotes by high-density variation within the MHC. To test this, we identified 14,787 variants across the genome that strictly differed between the six M1/M1 and the M1/M3 animals and used 50 kb bins to plot variant density across the genome (Figure 3A). As expected, we found that the highest density of variation marked the MHC on macaque chromosome 4. This proof of concept analysis identified a region of high variant density on chromosome 10, outside of the MHC, which we were unable to reconcile. To reduce the rate of false discovery, and to narrow our downstream analyses, we restricted subsequent analyses to candidate regions (‘islands’ of co-segregating variants) marked by the highest 5% of genome-wide variant densities.

### Identifying candidate control-modifying loci

**Figure 4:**
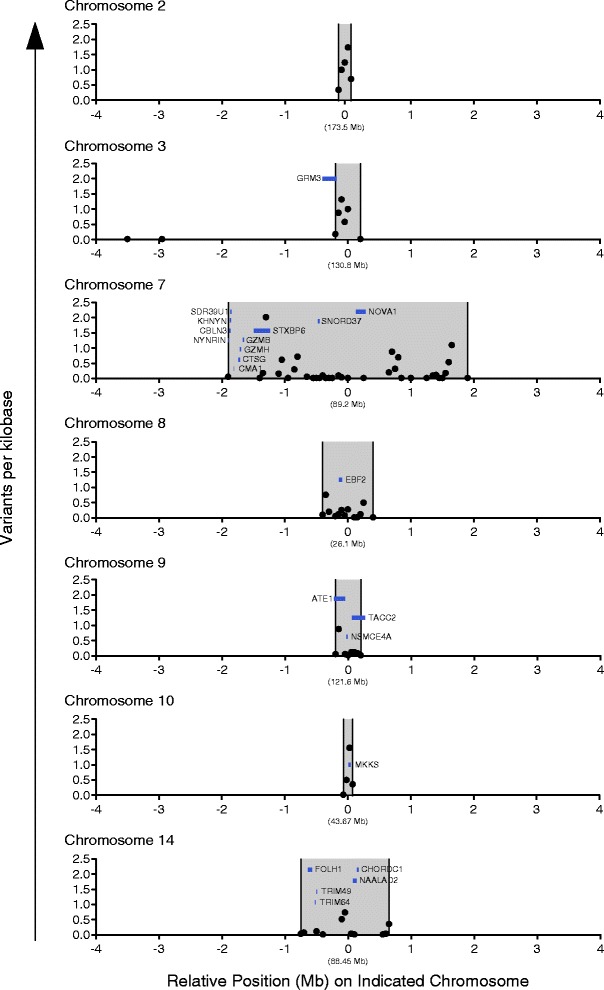
**Identification of candidate SIV control-modifying loci.** Whole genome sequencing identified seven regions marked by control-segregating variant densities meeting a 95th percentile cutoff (the highest 5% of genome-wide variant density) on macaque chromosomes 2, 3, 7, 8, 9, 10, and 14. Plots depict variant densities (shown in black) across each of the candidate regions with Ensembl gene annotations overlaid (shown in blue). The x-axes show distance (in megabases) relative to the center of each of the control-segregating regions.

We next applied our haplotype-level analysis to Group 1 and Group 2 animals in order to identify genetic variation that modified MHC-associated control. Across the genome, we identified 1,819 variant sites that strictly differed between Group 1 and Group 2, and plotted their density across the genome (Figure 3B). As stated above, to reduce the rate of false discovery in downstream analyses, we defined candidate CML as being marked by the 95th percentile of variant densities. We identified seven candidate CMLs across chromosomes 2, 3, 7, 8, 9, 10, and 14 (Figure 4). To determine the potential role of these seven loci in modifying MHC-associated control, we overlaid these variant-dense regions with Ensembl gene annotations [27] (Table 2), and manually queried the ImmPort database [28] to identify genes with known immunological function. Interestingly, the candidate CML on chromosome 7, which was marked by the highest density of control-segregating variants across the genome, was the only candidate that contained immune-related genes involved in T-cell function (Table 2). One of these genes, granzyme B (*gzmb*), codes for a key component of the cytotoxic granule machinery required for CD8-mediated lysis of target cells. Additionally, high-level expression and rapid degranulation of cytotoxic granule proteins, such as granzyme B, by epitope-specific CD8(+) T cells is associated with the maintenance of control during chronic HIV and SIV infection [13,16].

**Table 2:** **Genes within candidate control-modifying loci**

**Chromosome**	**Gene**	**Ensembl coordinates [** 27 **]**	**Length (bp)**	**Variants**	**T-cell function [** 29 **]**
3	*GRM3*	130400561-130623515	222,954	-	-
7	*NYNRIN*	87296239-87307068	10,829	-	-
	*CBLN3*	87324653-87327636	2,983	-	-
	*KHNYN*	87328804-87336472	7,668	-	-
	*SDR39U1*	87337848-87340858	3,010	-	-
	*CMA1*	87390195-87392979	2,784	-	-
	*CTSG*	87449456-87452155	2,699	-	-
	*GZMH*	87479687-87482876	3,189	-	+
	*GZMB*	87509950-87513263	3,313	-	+
	*STXBP6*	87705567-87964506	258,939	111	-
	*SNORD37*	88705465-88705530	65	-	-
	*NOVA1*	89330662-89479984	149,322	1	-
8	*EBF2*	25963548-26009524	45,976	3	-
9	*ATE1*	121374357-121553580	179,223	47	-
	*NSMCE4A*	121573109-121591667	18,558	2	-
	*TACC2*	121664364-121871454	207,090	12	-
10	*MKKS*	43683348-43712508	29,160	34	-
14	*FOLH1*	87860094-87925735	65,641	-	-
	*TRIM64*	87975789-87981217	5,428	-	-
	*TRIM49*	87997993-88004315	6,322	1	-
	*NAALAD2*	88578293-88633487	55,194	3	-
	*CHORDC1*	88642339-88664787	22,448	-	-

Among the seven candidate loci identified through whole genome sequencing analysis, six candidates contained coding genes (as determined using Ensembl annotations). Across these six regions, a total of 22 genes were identified, 9 of which contained control-segregating variants. For these 22 genes, the corresponding chromosome, coordinates, gene length, and number of control-segregating variants occurring within the gene are shown.

### Variation in granzyme B and M1-associated control of SIV

In order to determine whether variation in *gzmb* modifies SIV control, we began by defining alleles for the gene on the basis of one or more nucleotide differences in the 5′ and 3′ untranslated regions (UTRs), exons, and introns. Using this criterion, we identified a total of six *gzmb* alleles within MCM (Figure S2A in Additional file 2). Five animals in Group 1 shared the *Mafa-GzmB-01:01:01* allele (designated 'G1'), which was entirely absent from Group 2 animals (Figure S2B in Additional file 2). Additionally, we found that upon inclusion of additional M1(+) animals, the association between G1 and control of SIV replication remained intact (Figure S2C in Additional file 2). In addition to intronic and synonymous differences, G1 differs from the other alleles by a conservative non-synonymous polymorphism (lysine-to-arginine) that is shared with the G2 allele, which we did not find to be associated with SIV control. Although this substitution is located near a cluster of positively charged amino acids within GZMB implicated in cytotoxicity [30], it was not private to controllers (that is, it was also found in some SIV progressors) and is thus unlikely to underlie differential MHC-associated SIV control.

In the absence of compelling coding variation that might alter GZMB activity, we asked whether inheritance of G1 correlated with a difference in GZMB expression between Group 1 and Group 2 animals. We used flow cytometry to measure GZMB expression levels (median fluorescence intensity) in peripheral CD8(+) cells at pre-infection and acute infection time-points. We found higher GZMB expression by natural killer (NK) cells (CD8(+)CD3(-)), but not by T cells (CD8(+)CD3(+)), in G1(+) macaques prior to infection (*P* = 0.0163) and at the peak of virus replication on day 14 post-challenge (*P* = 0.0167) (Figure S2D in Additional file 2).

**Figure 5:**
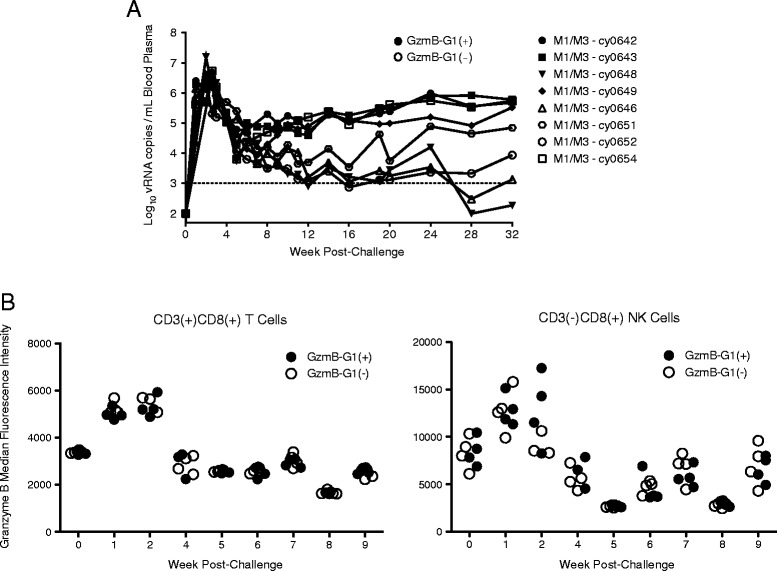
**Longitudinal viral loads and granzyme B expression for Cohort B.** The animals were followed for 32 weeks post-challenge. **(A)** Longitudinal viral loads are plotted for all eight animals. Data-points corresponding to G1(+) animals are indicated by shaded symbols, and G1(-) animals are indicated by open symbols. **(B)** Intracellular staining for granzyme B was performed to quantify expression levels of granzyme B over time. Longitudinal median fluorescence intensity is shown for both CD3(+)CD8(+) T cells (left panel) and CD3(-)CD8(+) NK cells (right panel). G1(+) animals are indicated by shaded symbols and G1(-) animals are indicated by open symbols.

Given our retrospective observations, we sought to prospectively test whether M1(+)G1(+) macaques control chronic SIV replication at a higher frequency than M1(+)G1(-) macaques. To test this hypothesis, we assembled an MHC-identical cohort (Cohort B) of eight M1/M3 female macaques (Table 3), composed of four G1(+) animals and four G1(-) animals. All eight animals became infected following a single intrarectal challenge with 7,000 TCID50 of SIVmac239. Viral loads for Cohort B animals began to diverge between 8 and 12 weeks post-challenge (Figure 5A). Throughout the first 20 weeks of infection, one out of four G1(+) animals, compared with three out of four G1(-) animals, demonstrated measurable post-peak control of SIV. Given the early indication that the G1(+) group would fail to demonstrate an increased frequency of SIV control, the study was terminated at 32 weeks post-challenge. At 32 weeks post-challenge, two animals, one G1(+) and one G1(-), exhibited durable maintenance of viral suppression. Altogether, we found no statistically significant difference in 32-week post-challenge viral loads between the G1(+) and G1(-) groups (*P* = 0.7101).

**Table 3:** **Demographics of animals used in prospective challenge study**

**ID**	**MHC**	**GZMB**	**32 week viral load (log10)**	**Sex**
cy0642	M1/M3	G1/G2	5.7	F
cy0643	M1/M3	G1/G2	5.8	F
cy0646	M1/M3	G2/G5	3.1	F
cy0648	M1/M3	G1/G4	2.3	F
cy0649	M1/M3	G1/G2	5.5	F
cy0651	M1/M3	G2/G3	4.9	F
cy0652	M1/M3	G4/G4	3.9	F
cy0654	M1/M3	G2/G5	5.7	F

Eight MHC-identical (M1/M3) Mauritian cynomolgus macaques were assembled into a cohort to prospectively test the influence of granzyme B genetics on MHC-associated control of SIV replication. Animal identifier, MHC genotype, 32 week viral loads (log_10_ viral RNA copies per milliliter of plasma), and sex are shown for each animal. The granzyme B genotype for each animal is shown.

Additionally, we used flow cytometry to measure *ex vivo* GZMB expression levels within peripheral CD8(+) cells at multiple acute infection time-points (Figure 5B). In contrast to our retrospective observations, we found no statistically significant difference in GZMB expression levels within NK cells (CD8(+)CD3(-)) or T cells (CD8(+)CD3(+)) between G1(+) and G1(-) animals. Thus, despite our retrospective observations, we were unable to prospectively confirm an association between inheritance of G1 and higher expression of GZMB during acute SIV infection.

### Comparison to human genome-wide association studies

In order for animal models of AIDS virus control to contribute to the understanding of human control of HIV, the host processes underlying control need to be shared. Despite the MHC being the major determinant of control in both humans and macaques, it is not yet clear whether minor determinants, or modifiers of the major determinant, of control exist, and if they are conserved. We sought to determine whether genomic variants associated with either control or acquisition of HIV corresponded to the candidate CML that we identified through our macaque analysis. We determined the human coordinates that corresponded to the candidate CML on macaque chromosomes 2, 3, 7, 8, 9, 10, and 14. Upon surveying the International HIV controller study [1] and the International HIV Acquisition Consortium (IHAC) [31], we did not identify any variants that met the rigorous threshold for genome-wide statistical significance.

## Discussion

Although there have been recent hints at success [32–34], the majority of attempts to immunize against HIV have failed to demonstrate protective efficacy. However, rare spontaneous chronic phase control of demonstrably pathogenic AIDS virus replication provides evidence that the host immune response is able to control HIV and SIV replication. The strongest immunological correlate of this control is host inheritance of at least one of a handful of specific MHC class I alleles. However, MHC-associated suppression of viral replication is incompletely penetrant, which indicates an opportunity to identify additional host genetic variation that modifies the MHC's protective effect.

Identifying modifiers of MHC-associated HIV control in humans is confounded by multiple factors. First, the heterogeneity of circulating HIV means that no two individuals are infected with the same virus, with the same sequence or predilection to cause disease. Second, since the symptoms of acute HIV infection are unremarkable, few such cases present clinically. Lastly, an individual’s ability to maintain control of HIV is likely influenced by the durability of immune containment, which is mediated by processes that begin to act during early infection. Since poor containment during early chronic infection precedes the loss of control of viral replication, proper assessment of genetic effects on durable virus control necessitates studying infection longitudinally, which is not practical in humans.

Asian-origin macaques, such as rhesus and cynomolgus macaques, are susceptible to SIV, and exhibit incompletely penetrant MHC-associated control of virus replication. Having been deposited on the island of Mauritius within the past half-millennia, MCMs represent an exceptional outbred population of Asian-origin macaques that experienced a strong genetic bottleneck or founder effect. Their restricted genetics makes them ideal for testing the broad hypothesis that genetic variation outside of the MHC modifies MHC-associated control of AIDS virus replication. Moreover, the haplotype sharing we demonstrate here for SIV control is extensible to identifying genetic correlates of other variable host responses to infectious disease, pharmaceuticals, and transplantation.

Correlating minor genetic determinants of AIDS virus control with immunologic effector mechanisms may elucidate measurable correlates of protection that can be used to assess the efficacy of therapeutic and prophylactic HIV vaccine candidates. Since the selection of interferon-gamma [35] as the primary readout of T cell functionality in vaccine studies is not based on any specific, functional role in effective T-cell responses in individuals who spontaneously control HIV or SIV, incorporating measures of other immune effector molecules, whose genetics underlie differences in viral containment, might well inform studies of vaccine efficacy.

While we believe our results serve as the most comprehensive interrogation of genetic variation segregating AIDS virus controllers from progressors, they are subject to the limitations inherent to mapping to highly complex reference genomes, and are highly dependent on the completeness and accuracy of reference annotations. The rheMac2 draft of the rhesus macaque reference genome was completed in 2006 and consequently did not benefit from recent advances in genome sequencing and annotation [36]. Refined rhesus macaque and cynomolgus macaque genome sequences, with empiric gene annotations informed by transcript sequencing, are currently in development. We anticipate that re-analysis of this dataset against newer genomes may be informative, particularly given the genetic dissimilarities between cynomolgus and rhesus macaques [37,38], such as in complex regions or in regions that are otherwise poorly resolved in the rheMac2 assembly. Segmental duplications or copy number variation is notoriously difficult to assemble during initial genome assembly, and such complex regions may harbor genes that influence viral control. For example, our analysis was unable to identify and resolve differences in genome structure (for example, large insertions, inversions, and translocations) that exist between Mauritian-origin cynomolgus macaques and Indian-origin rhesus macaques. Additionally, although Cohort A was composed of all male animals, we were unable to assess variation on the Y chromosome due the rheMac2 reference genome being based on a female rhesus macaque.

Our approach identified regions marked by high-density control-segregating variation, which we designated candidate control-modifying loci. Surprisingly, across the seven candidates that we identified, only one of these regions comprised genes with known immunological function. This region, found on chromosome 7, was marked by the highest density of control-segregating variation and contained genes implicated in cellular immunity such as those encoding cathepsin G, granzyme B, granzyme H, and chymase A. Previous studies have demonstrated differences in virus-specific CD8(+) T-cell cytotoxicity between progressors and controllers [13,15,16], which made genetic variation within or surrounding genes encoding cytotoxic granule proteins compelling candidate modifiers of SIV control. Granzyme B is one of the hallmark cytotoxic granules required for cognate lysis of virus-infected cells by CD8(+) effector cells, which strongly suggested that our genome-wide analysis complemented the functional correlations identified by other groups. We were able to identify an allele of granzyme B, designated G1, that correlated with SIV control in both the whole genome sequencing cohort and a combined cohort that included additional SIV-infected MCMs (Figure S2C in Additional file 2). Additionally, we found that G1(+) animals expressed higher levels of GZMB in NK cells during early infection. However, we were unable to confirm a role for granzyme B variation in MHC-associated SIV control upon prospectively challenging a cohort of MHC-identical *gzmb*-defined animals.

There are several possible explanations for our discrepant results, but a few merit specific attention. First, the association that we identified between chromosome 7 variation and viral control may have been spurious. Given that any combination of six animals will share some number of variants, our analysis cannot distinguish spurious association results from variants that causally modify MHC-associated control. However, future genomic analyses of SIV control in MCMs and other non-human primates will serve to confirm and narrow down regions of the genome that contain authentic control-modifying variation. Second, it is conceivable that we simply selected the wrong control-modifying loci to test prospectively. We selected the candidate CML on chromosome 7 based upon it having the highest density of control-segregating variation across the genome and it being the only candidate to comprise genes with established immune-related function. It is important to note that we did not implement formal statistical testing or power calculations in this exploratory assessment of host genetic variation in SIV control, but as genomic data for additional animals become available, candidate gene discovery will benefit from such validation.

Lastly, although we attempted to control for as many potentially confounding variables as possible when designing our prospective challenge study (for example, virus stock, dose, and route of challenge), a major variable that was not held consistent between the retrospective cohort (all male macaques) and the prospective cohort (all female macaques) was the sex of the animals. In our experience, female Indian-origin rhesus and Mauritian-origin cynomolgus macaques experience higher levels of SIVmac239 replication than their MHC-matched male contemporaries. This anecdotal difference is complementary to a body of literature on sex-specific differences in immunological function (reviewed in [39–43]). It is desirable to assume that an efficacious HIV vaccine will exploit an immunological process to universally confer protective immunity. However, the combined results from our retrospective and prospective studies anecdotally suggest that sex differences may modulate AIDS virus control and, as a recent National Institutes of Health policy change has mandated [44], must be considered when designing and evaluating genomic studies.

## Conclusion

We provide the first whole genome sequencing study on host control of AIDS virus replication. We confirmed that whole genome sequencing could be used to identify phenotype-associated genetic variation by assessing differences between two groups of MHC-defined animals, one group of six MHC homozygotes and one group of six MHC heterozygotes. We further found that binning variants into 50 kb bins and plotting variant density across the genome accurately identified genomic regions containing genetic variation that differentiated these *a priori* MHC-defined animals. We then applied this approach to evaluate SIV controllers and progressors that shared a protective MHC haplotype [9]. Through this analysis, we identified seven regions across the macaque genome that encompassed high-density control-segregating variation. We conclude that whole genome sequencing can be used to identify genetic variation that distinguishes groups of phenotypically different individuals. Using the MCM model, such differences can be tested prospectively for causality.

## Materials and methods

### Animals, viral infections, and viral loads

As part of a previous animal study [9,45], 18 male MCMs, including the 12 Cohort A animals, were infected following a single intrarectal challenge with 7,000 TCID50 of the molecularly cloned SIVmac239 virus [GenBank: M33262]. Similarly, 8 female MCMs that comprised Cohort B were infected following a single intrarectal challenge with 7,000 TCID50 of SIVmac239. For all 26 animals, SIV viral loads were quantified using a previously described viral load assay [46]. All animals used in this study were cared for by the staff at the Wisconsin National Primate Research Center according to regulations and guidelines of the University of Wisconsin Institutional Animal Care and Use Committee. Details of this study (UW-Madison Animal Care and Use Protocol No. G00517) were approved by the University of Wisconsin Institutional Animal Care and Use Committee, in accordance with recommendations of the Weatherall report.

### DNA library preparation

Blood was drawn from 18 MCMs, and peripheral blood mononuclear cells (PBMCs) were prepared, and genomic DNA was isolated. Genomic DNA was then quantified and quality checked using PicoGreen and gel analyses, and subsequently used to construct Illumina paired-end libraries according to the manufacturer’s protocol (Illumina part number 1005361, revision D) with the modifications described below. A more complete description is available at [47]. Libraries were prepared using Beckman robotic workstations (Biomek FX and FXp models; Beckman Coulter Inc., Brea, CA, USA). Briefly, 1 μg of genomic DNA was sheared into fragments of approximately 300 to 400 bp with the Covaris E210 system (Covaris Inc., Woburn, MA, USA). The sheared DNA was then end-repaired, A-tailed and ligated to Illumina multiplexing paired-end adaptors. Ligation-mediated PCR was performed for six to eight cycles of amplification using the 2X SOLiD Library High Fidelity Amplification Mix (a custom product manufactured by Invitrogen (Carlsbad, CA, USA)). Universal primer IMUX-P1.0 and a pre-capture barcoded primer were used in the PCR amplification. In total, 18 such barcodes were used on these samples. Purification was performed with Agencourt AMPure XP beads (Beckman Coulter Inc., Brea, CA, USA) after enzymatic reactions, and following the final purification, quantification and size distribution of the ligation-mediated PCR product was determined using the LabChip GX electrophoresis system (PerkinElmer, Melville, NY, USA).

### Genome sequencing

Library templates were prepared for sequencing using Illumina’s cBot cluster generation system with TruSeq PE Cluster Generation Kits (Illumina catalog number PE-401-3001) according to the manufacturer’s protocol. Briefly, these libraries were denatured with sodium hydroxide and diluted to 6 to 9 pM in hybridization buffer in order to achieve a load density of approximately 800 K clusters/mm^2^. Each library was loaded in three lanes of a HiSeq flow cell, and each lane was spiked with 2% phiX control library for run quality control. The sample libraries then underwent bridge amplification to form clonal clusters, followed by hybridization with the sequencing primer. Sequencing runs were performed in paired-end mode using the Illumina HiSeq 2000 platform. Using the TruSeq SBS Kits (Illumina catalog number FC-401-3001), sequencing-by-synthesis reactions were extended for 101 cycles from each end, with an additional seven cycles for the index read. Sequencing runs generated approximately 350 to 500 million filter-pass reads on each flowcell lane, yielding an average of 44 Gb per lane. On average, 118 Gb of unique aligned sequence was generated per sample.

### Illumina analysis and variant calls

Initial analysis of Illumina sequence reads was performed using the Baylor College of Medicine (BCM)-HGSC Mercury analysis pipeline [48]. This pipeline addresses all aspects of data processing, moving data stepwise through various analysis tools from the initial sequence generation on the instrument to the generation of alignments (BAM files). There are four main steps in this pipeline. First Illumina’s software is used to perform base calling and filter out low quality reads. These reads were then aligned to rheMac2 using the Burrows-Wheeler aligner (BWA) [49], and Picard tools was used to mark PCR duplicates and to aggregate alignments and generate a single BAM file for each animal. These alignments were deposited into the NCBI Sequence Read Archive (SRA), and are accessible through BioProject [BioProject: PRJNA257343], or SRA [SRA: SRP045278].

SAMtools v0.1.17 [50] was used to call variants, including substitutions and small indels, and to produce a variant call file (VCF). The caller only used reads with mapping quality greater than 10, and did not apply additional filters in order to maximize sensitivity. Next, annotation data were added to the VCF using SnpEff v3.3 [51]. Per-sample read depths (RDPs) were computed using custom scripts. Next, snpSift v3.6 [52] was used to filter on variants supported by at least 10 reads for all 18 animals (RDP >9), and that met a quality threshold of at least 30 (Q ≥30). To generate a population-level VCF for use in downstream analyses, snpSift v3.6 [52] was used to exclude variants corresponding to species-specific differences between the MCM and the rheMac2 reference sequence, which were identified as homozygous variants present in all animals.

### Genome-wide coverage analysis and mapping statistics

To generate coverage plots, BEDTools genomecov [27] was used to extract the read depth of all genomic positions from each sample’s BAM file. Next, arithmetic mean read depths were calculated across the genome using 10 kb bins, and plots were generated using a custom tool (source code available at [28]). Additionally, the total number of reads mapping to rheMac2 were calculated by running SAMtools flagstat [50] on each sample’s BAM file.

### Evaluation of heterozygous variation within the MHC of short tandem repeat-typed MHC homozygotes and heterozygotes

Using the population-level VCF, snpSift v3.6 [52] was used to generate animal-specific chromosome 4 VCFs for 18 of the animals, including six M1 homozygotes, six M1/M3 animals, and six M3 homozygotes. Next, each animal-specific VCF was filtered using snpSift v3.6 [52] to remove homozygous variants prior to being loaded into the VCFtools [53] SNPdensity tool in order to calculate the density of heterozygous variants, using 10 kb bins, across macaque chromosome 4. Prism v5.0 (GraphPad, La Jolla, CA) was used to plot heterozygous variant density spanning the MHC for each animal (Figure 2).

### Calculation of group-segregating variant densities and generation of gene overlay plots

To identify variants that differentiated the six M1/M1 animals from the six M1/M3 animals, the population-level VCF was filtered using snpSift v3.6 [52] to preserve sites that matched the reference in all six M1/M1 animals and variant in all six M1/M3 animals (and the reciprocal). Once generated, this M1/M1 versus M1/M3 VCF was manually sorted and curated to exclude variants that corresponded to non-canonical chromosomes in the rheMac2 genome (for example, chr4_random). Identical methodology was used to identify sites that strictly differed between all Group 1 animals (controllers) and all six Group 2 animals (progressors). In order to calculate and plot group-segregating variant densities (corresponding to both substitutions and short indels), these group-specific VCFs were fed into the VCFtools SNPdensity program [53]. Genome-wide variant density was calculated using 50 kb bins to assess differences between M1/M1 and M1/M3 animals (Figure 3A) and between Group 1 and Group 2 animals (Figures 3B and 4). Downstream analyses were focused on regions of the macaque genome marked by the 95th percentile of genome-wide variant density (or the highest 5% of variant density across the genome). Gene annotations were downloaded from the rhesus macaque (Mmul_1) assembly in release 75 of Ensembl [54] in order to generate the gene overlays for the variant density plots corresponding to the seven candidate control-modifying loci (Figure 4). Genes contained within candidate CMLs were determined to have immune-related function by manually querying the ImmPort database [29]. Gene overlay plots were created using GraphPad Prism v5.0.

### Comparison to human GWASs

In order to determine whether human genomic regions corresponding to the candidate macaque CMLs were implicated in HIV disease, we interrogated the Ricopili database [55]. This disease-focused database aggregates results from various GWAS, in this case, the International HIV controller study [1] and the International HIV Acquisition Consortium (IHAC) [31]. We obtained the *P*-values for single-nucleotide polymorphisms within the corresponding regions, and found that none of them reached the GWAS statistical threshold for significance (*P* <5E-8).

### Granzyme B typing by sequencing

Briefly, genomic DNA was isolated and used as template for *gzmb* amplification. For PCR, the forward primer (5′-GGGCAGCATTCACAGAAA-3′) and the reverse primer (5′-CCTGCATAGCACAGAACTGG-3′) were used to amplify a 3.4 kb amplicon corresponding to the region of the rhesus macaque genome (chr7:87,509,903-87,513,329) containing the *gzmb* gene. Amplification was performed using Phusion HF Master Mix (Thermo Scientific, Waltham, MA, USA), in a total reaction volume of 25 μl. Reactions were cycled as follows: 98°C for 2 minutes followed by 35 cycles of 98°C for 10 s, 62°C for 15 s, 72°C for 3 minutes. A final extension was performed at 72°C for 10 minutes, and the reaction was held at 10°C until being run on an agarose gel. Bands corresponding to the 3.4 kb product were extracted and Illumina compatible libraries were generated using the Illumina Nextera XT kit (catalog number FC-131-1024). Sequencing libraries were cleaned using the Agencourt Ampure XP system (catalog number A63882) and subsequently sequenced on the Illumina MiSeq. On average, 74,000 reads per sample were generated. Low quality (Q <30) and short reads (<100 bp) were removed and the remaining reads were mapped against the rhesus macaque *gzmb* reference sequence. Variants in *gzmb* were phased using Beagle v4.0 [56], and six alleles were defined.

### Evaluating granzyme B expression

For Cohort A, cryopreserved PBMCs were thawed at 37°C and washed once in R10 media prior to staining. As samples permitted, 1 to 2 million PBMCs were stained with 2 μl anti-CD3 Alexa Fluor 700 (clone SP34-2; BD Biosciences, San Jose, CA, USA) and 1 μl anti-CD8 Pacific Blue (clone RPA-T8; BD Biosciences) in 150 μl R10 for 30 minutes at room temperature. Cells were then washed with fluorescence-activated cell sorting (FACS) buffer, and fixed with 1% paraformaldehyde. Cells were fixed for 30 minutes at 4°C, and washed once with FACS buffer. Cells were permeabilized by adding 75 μl medium B (Life Technologies, Grand Island, NY, USA), and simultaneously stained for granzyme B with 1 μl anti-GZMB allophycocyanin (clone GB12; Life Technologies) for 30 minutes at room temperature. Cells were washed twice with FACS buffer, fixed in 1% paraformaldehyde, and placed at 4°C until being run on a BD-LSRII (BD Biosciences). Analysis was performed using FlowJo software (version 9.7.1, Tree Star, Ashland, OR, USA). This methodology was then used to assess GZMB expression in Cohort B, with the exception of staining freshly processed cells, and not cryopreserved cells.

### Data accessibility

Whole genome alignments to rheMac2 for all 18 retrospective cohort animals were deposited into the NCBI SRA as BAM files, and are accessible through BioProject [BioProject: PRJNA257343] or through SRA [SRA: SRP045278]. The six granzyme B alleles identified in this study have been deposited to GenBank and can be accessed through their corresponding accession numbers, as follows: *Mafa-GzmB:01:01:01* [GenBank: KM281203], *Mafa-GzmB:01:01:02* [GenBank: KM281207], *Mafa-GzmB:02:01:01* [GenBank: KM281204], *Mafa-GzmB:02:01:02* [GenBank: KM281205], *Mafa-GzmB:02:01:03* [GenBank: KM281206], and *Mafa-GzmB:02:01:04* [GenBank: KM281208].

## Additional files

Additional file 1: Figure S1. Whole genome sequencing coverage for Cohort A. Mapping coverage for each Cohort A animal is shown across 21 macaque chromosomes. The y-axis corresponds to the number of reads, and the x-axis corresponds to the position on the indicated chromosome. Gaps reflect regions in which reads did not map to the rhesus macaque (rheMac2) reference genome. Additional file 2: Figure S2. Granzyme B in Cohort A. **(A)** Six distinct alleles for *gzmb* were identified for MCMs. A single non-synonymous coding polymorphism (black bar) that differentiates two of the alleles from the other four. This conservative lysine-arginine mutation is accompanied by various synonymous (green bar) and intronic (blue bar) polymorphisms. At 52 weeks post-challenge, G1(+) animals had significantly lower viral loads than G1(-) animals, both within **(B)** Cohort A (*P* =0.0001; unpaired *t*-test; two-sided *P*-value), and **(C)** a cohort including 7 additional M1(+) animals (*P* =0.0002; unpaired *t*-test; two-sided *P*-value). **(D)** Flow cytometry was used to measure granzyme B expression within CD3(+)CD8(+) T cells (left panel) and CD3(-)CD8(+) NK cells (right panel). Median fluorescence intensity (MFI) for GZMB is displayed on the y-axis, and plotted for G1(+) animals (shaded symbols) and G1(-) animals (open symbols) at 0, 2, 3, 6, 8, 10, and 12 weeks post-challenge. Asterisks mark time-points at which the difference in granzyme B expression between G1(+) and G1(-) samples was statistically significant (*P* <0.05).

## Abbreviations

AIDS: acquired immune deficiency syndrome
bp: base pairs
CML: control-modifying locus
GWAS: genome-wide association study
HIV: human immunodeficiency virus
HLA: human leukocyte antigen
MCM: Mauritian cynomolgus macaques
MHC: major histocompatibility complex
NK: natural killer
PBMC: peripheral blood mononuclear cell
PCR: polymerase chain reaction
SIV: simian immunodeficiency virus
SRA: Sequence Read Archive
VCF: variant call format
