# Supplementary figures and images for: Whole genome sequencing of SIV-infected macaques identifies candidate loci that may contribute to host control of virus replication
Source: Genome Biol. 2014 Nov 7;15(11):478. doi: 10.1186/s13059-014-0478-z (PMC4223156; doi:10.1186/s13059-014-0478-z)

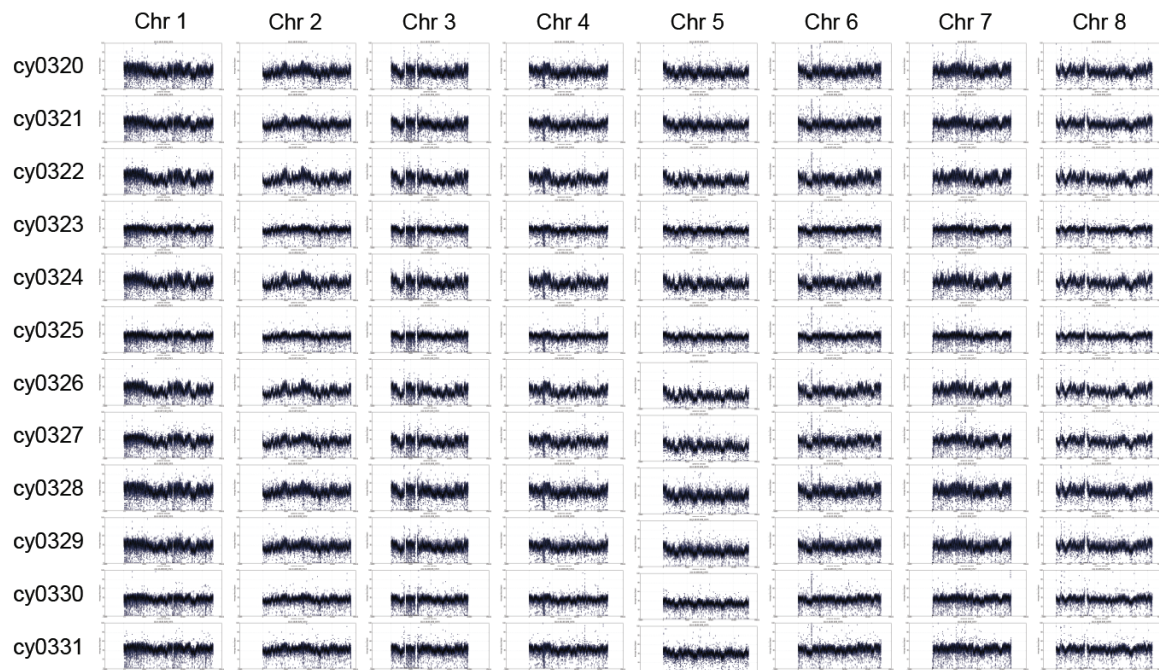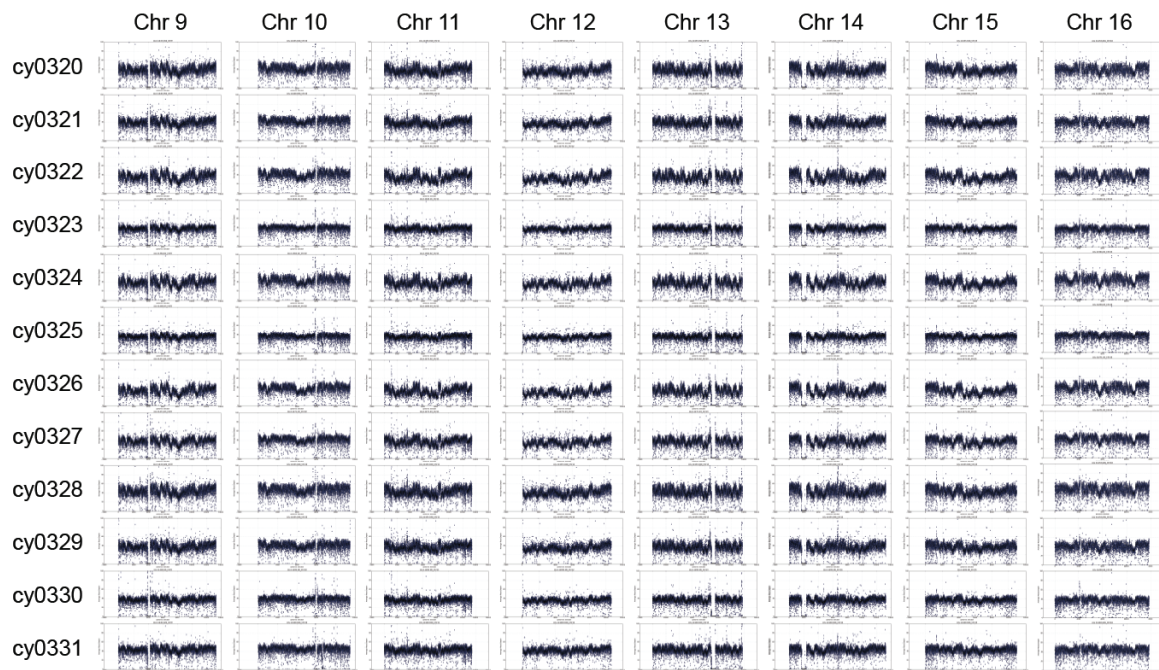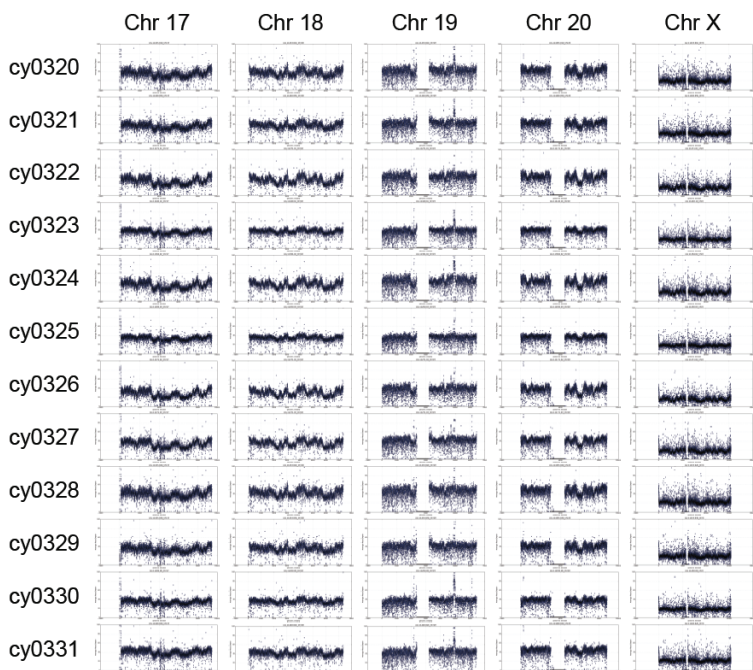

Supplement: Additional file 1: Figure S1. — Whole genome sequencing coverage for Cohort A. Mapping coverage for each Cohort A animal is shown across 21 macaque chromosomes. The y-axis corresponds to the number of reads, and the x-axis corresponds to the position on the indicated chromosome. Gaps reflect regions in which reads did not map to the rhesus macaque (rheMac2) reference genome. [file 13059_2014_478_MOESM1_ESM.pdf]

A

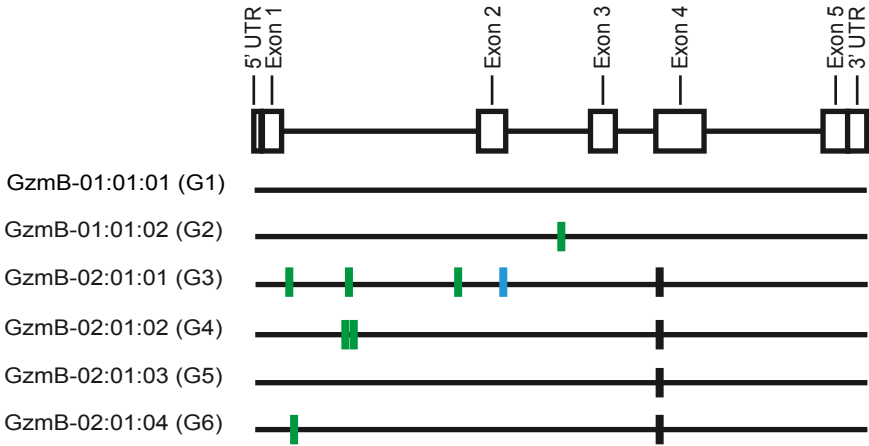

B

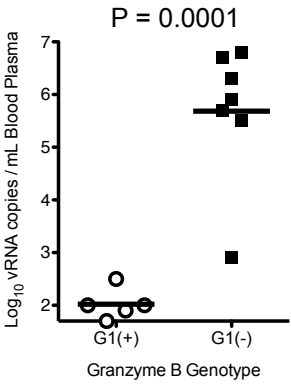

C

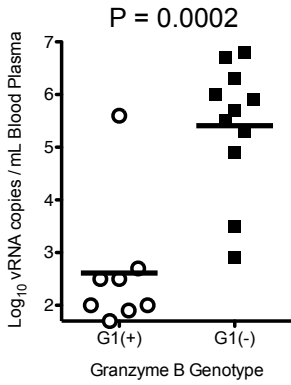

D

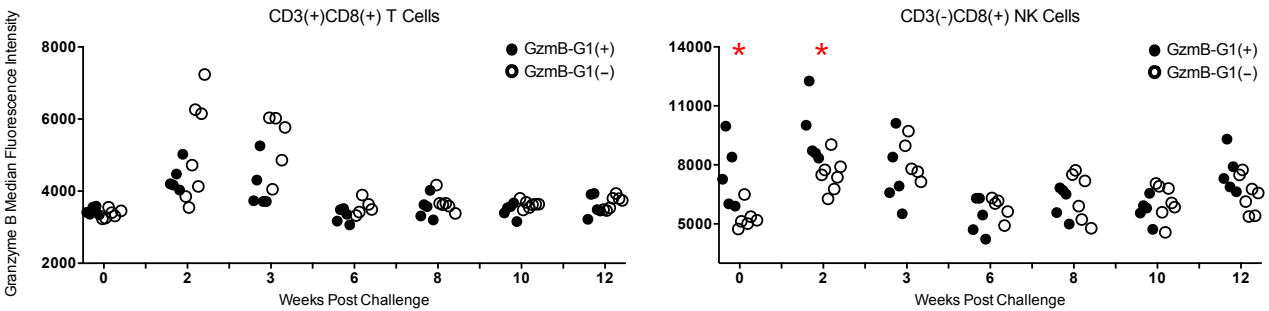

Supplement: Additional file 2: Figure S2. — Granzyme B in Cohort A. (A) Six distinct alleles for gzmb were identified for MCMs. A single non-synonymous coding polymorphism (black bar) that differentiates two of the alleles from the other four. This conservative lysine-arginine mutation is accompanied by various synonymous (green bar) and intronic (blue bar) polymorphisms. At 52 weeks post-challenge, G1(+) animals had significantly lower viral loads than G1(-) animals, both within (B) Cohort A (P =0.0001; unpaired t-test; two-sided P-value), and (C) a cohort including 7 additional M1(+) animals (P =0.0002; unpaired t-test; two-sided P-value). (D) Flow cytometry was used to measure granzyme B expression within CD3(+)CD8(+) T cells (left panel) and CD3(-)CD8(+) NK cells (right panel). Median fluorescence intensity (MFI) for GZMB is displayed on the y-axis, and plotted for G1(+) animals (shaded symbols) and G1(-) animals (open symbols) at 0, 2, 3, 6, 8, 10, and 12 weeks post-challenge. Asterisks mark time-points at which the difference in granzyme B expression between G1(+) and G1(-) samples was statistically significant (P <0.05). [file 13059_2014_478_MOESM2_ESM.pdf]
